# Supplementary material for: C1q and Tumor Necrosis Factor Related Protein 9 Protects from Diabetic Cardiomyopathy by Alleviating Cardiac Insulin Resistance and Inflammation
Source: Cells. 2023 Jan 29;12(3):443. doi: 10.3390/cells12030443 (PMC9914367; doi:10.3390/cells12030443)
Supplement: Supplementary file 1 [file cells-12-00443-s001.zip › cells-2132789-supplementary.pdf]

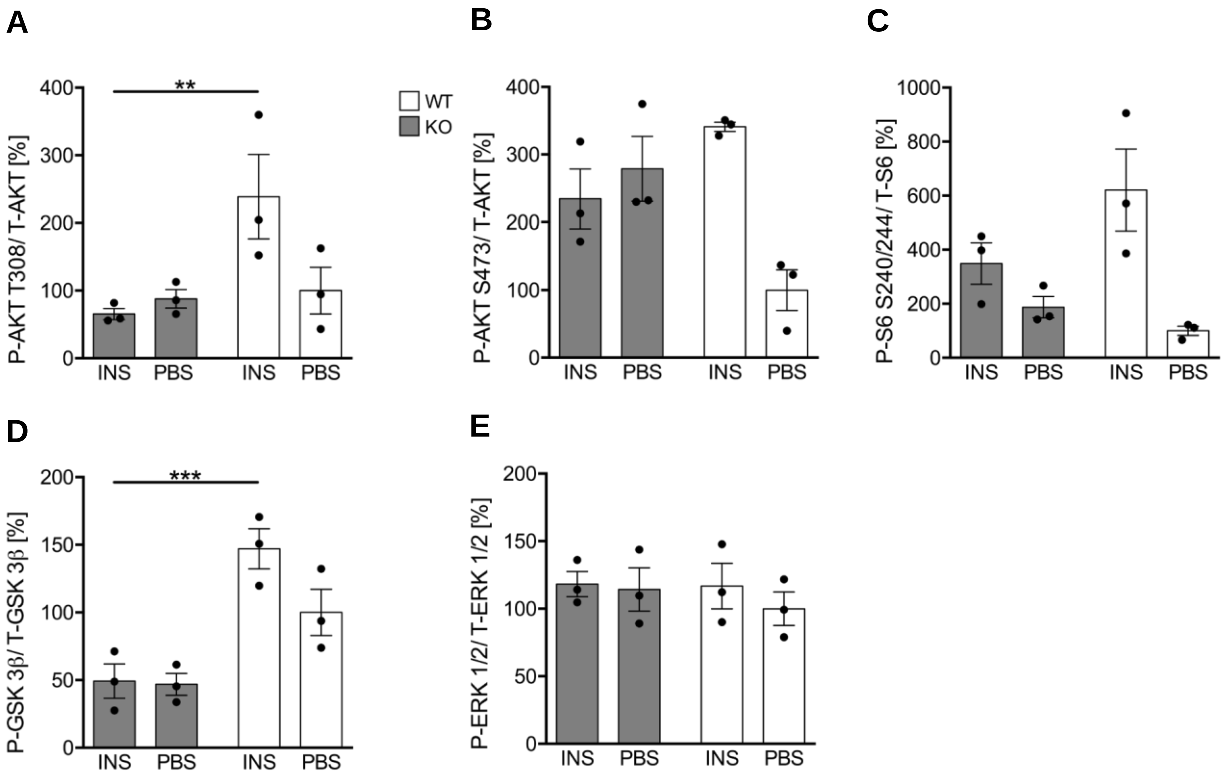

Supplemental Figure S1. CTRP9 knock-out mice (KO) do not activate Akt/Proteinkinase B dependent signaling in response to insulin. A-E Densitometric quantification of the Western blots of the indicated proteins that are shown in Figure 3D.

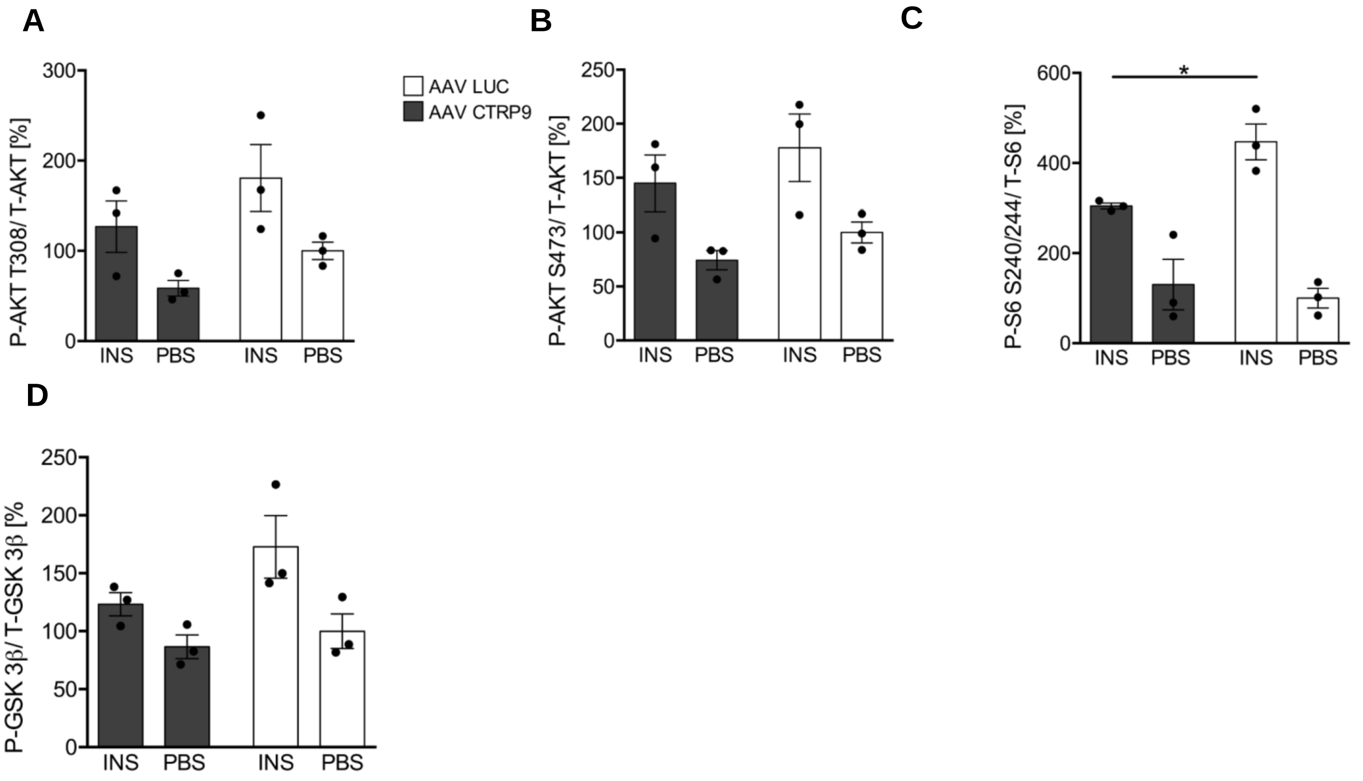

Supplemental Figure S2. Cardiac CTRP9 overexpression in mice does not change signaling in response to insulin. A-E Densitometric quantification of the Western blots of the indicated proteinsthat are shown in Figure 6C.

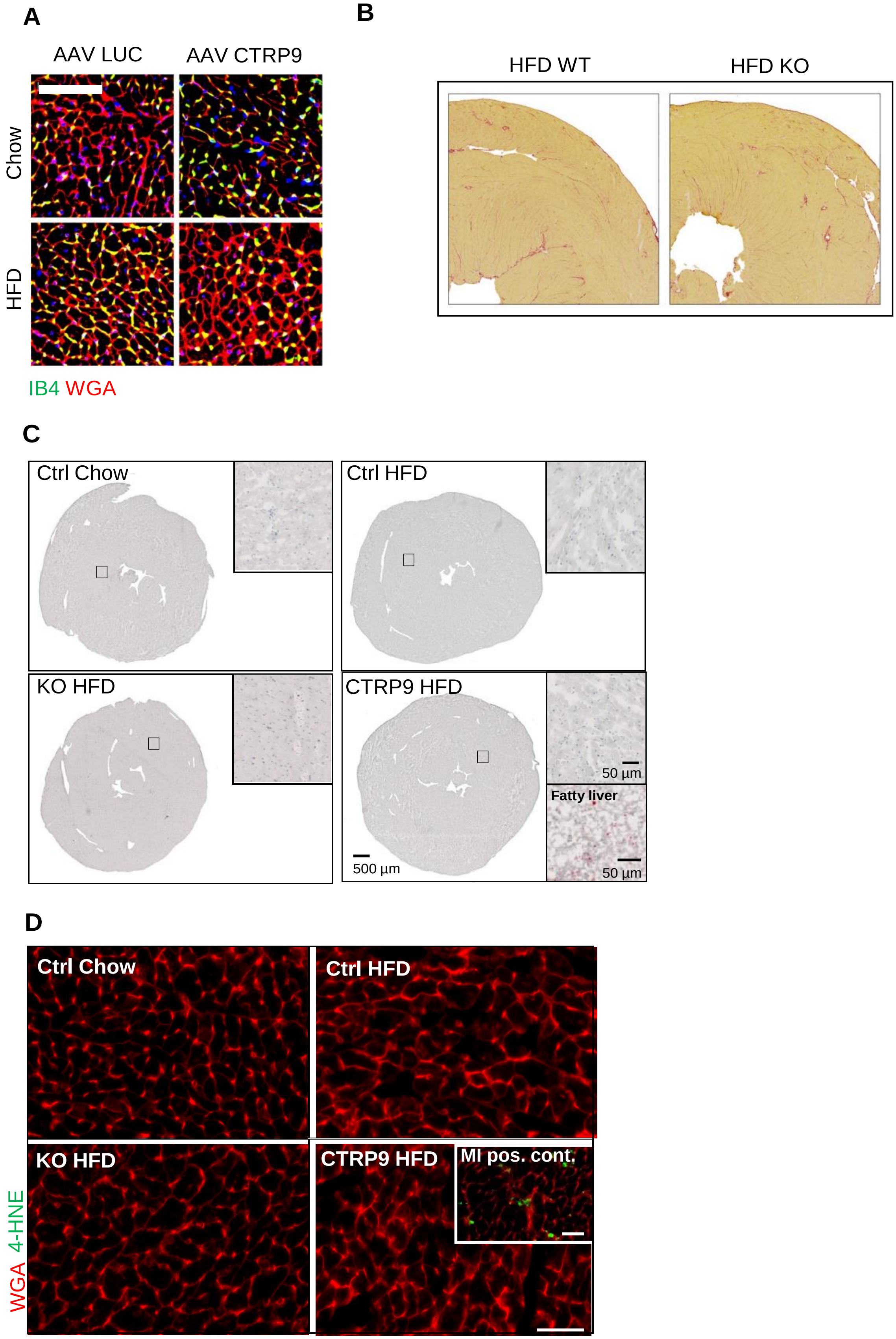

Supplemental Figure S3. Histological examinations in CTRP9 knock-out mice and in mice with cardiac CTRP9 overexpression. (A) Immunofluorescence staining for Isolectin B4 (IB4) and wheat-germ agglutinin (WGA) to measure cardiomyocyte cross-sectional area and myocardial capillary density in the indicated mice.Scale bar: 100µm. (B) Sirius red staining of myocardial sections of the indicated mice after high fat diet (HFD)exposure to analyze fibrosis. (C) Oil red O staining to analyze lipid deposition in heart sections from mice as indicated. Control mice (Ctrl) were wild-type mice with and without AAV-Luc administration. Fatty liver tissue served as positive control. (D) Immunofluorescence staining of heart sections of mice as indicated for WGA and 4-hydroynonenal (4-HNE) to analyze oxidative damage. Heart tissue from mice after myocardial infarction (MI) served as positive control. Scale bar: 50µm.

A

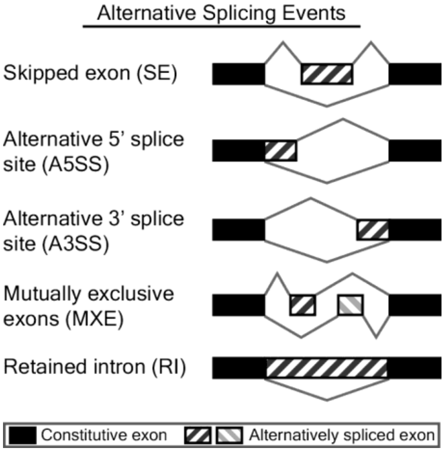

		HFD	
		WT	CTRP9 KO
EventType	SignificantEvents JC	SigEventsJC HigherInclusion	SigEventsJC HigherInclusion
**SE**	54	20	34
**A5SS**	7	4	3
**A3SS**	18	10	8
**MXE**	19	12	7
**RI**	34	25	9

Summary from RMATS, FDR≤0.05

B

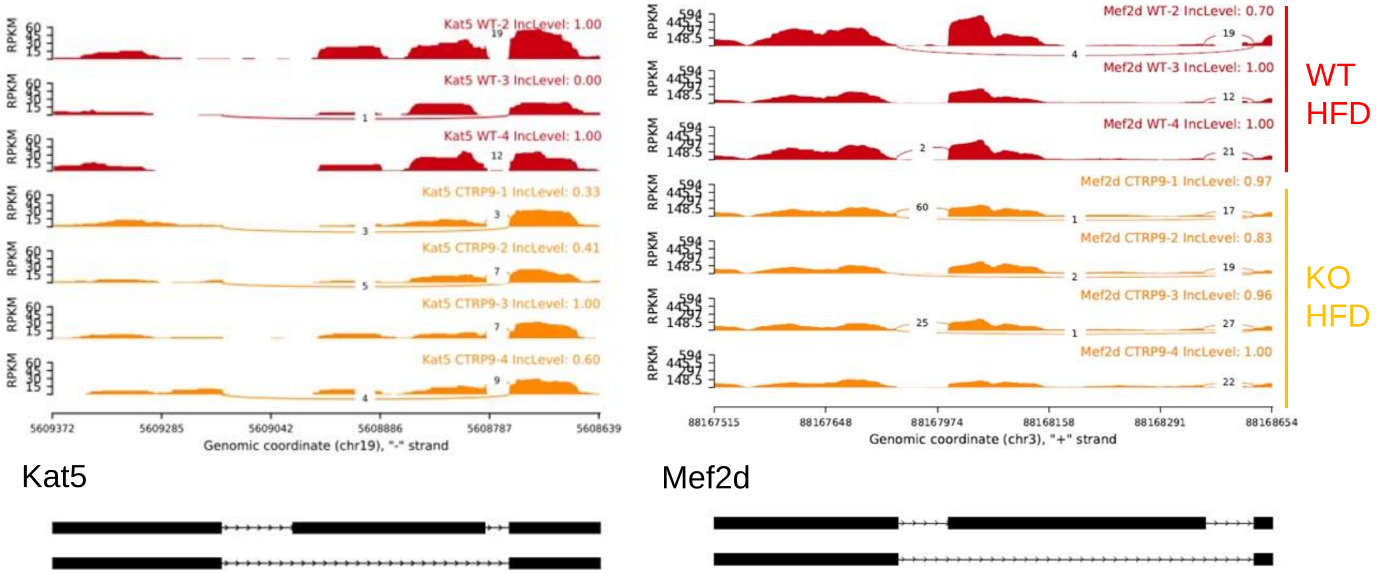

C

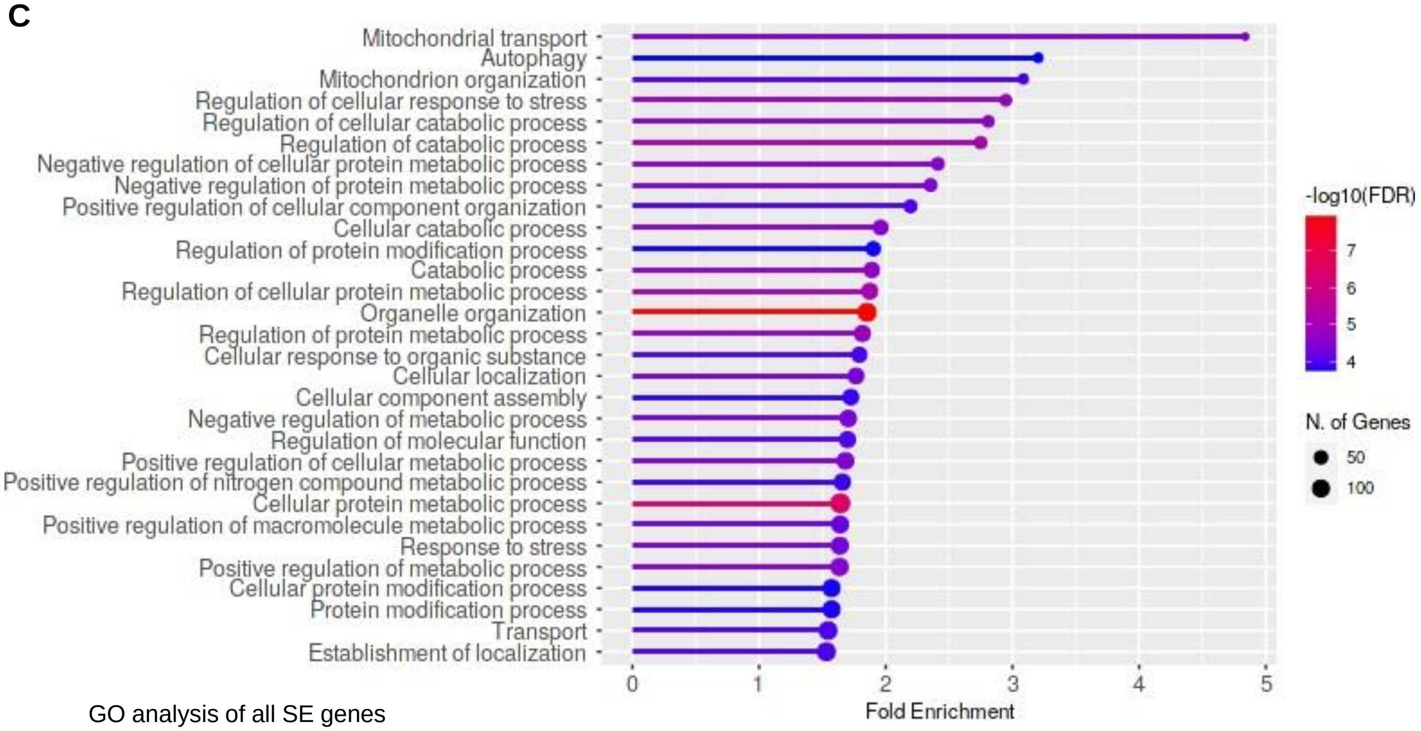

GO analysis of all SE genes

Supplemental Figure S4. Alternative splicing events in CTRP9 knock-out (KO) mice during high fat diet (HFD). (A) Different alternative splicing events in the indicated mice. (B) Example of alternative splicing in the *Kat5* and *Mef2d* genes in the indicated mice. (C) Functional classes of genes with skipped exons in CTRP9 KO mice.

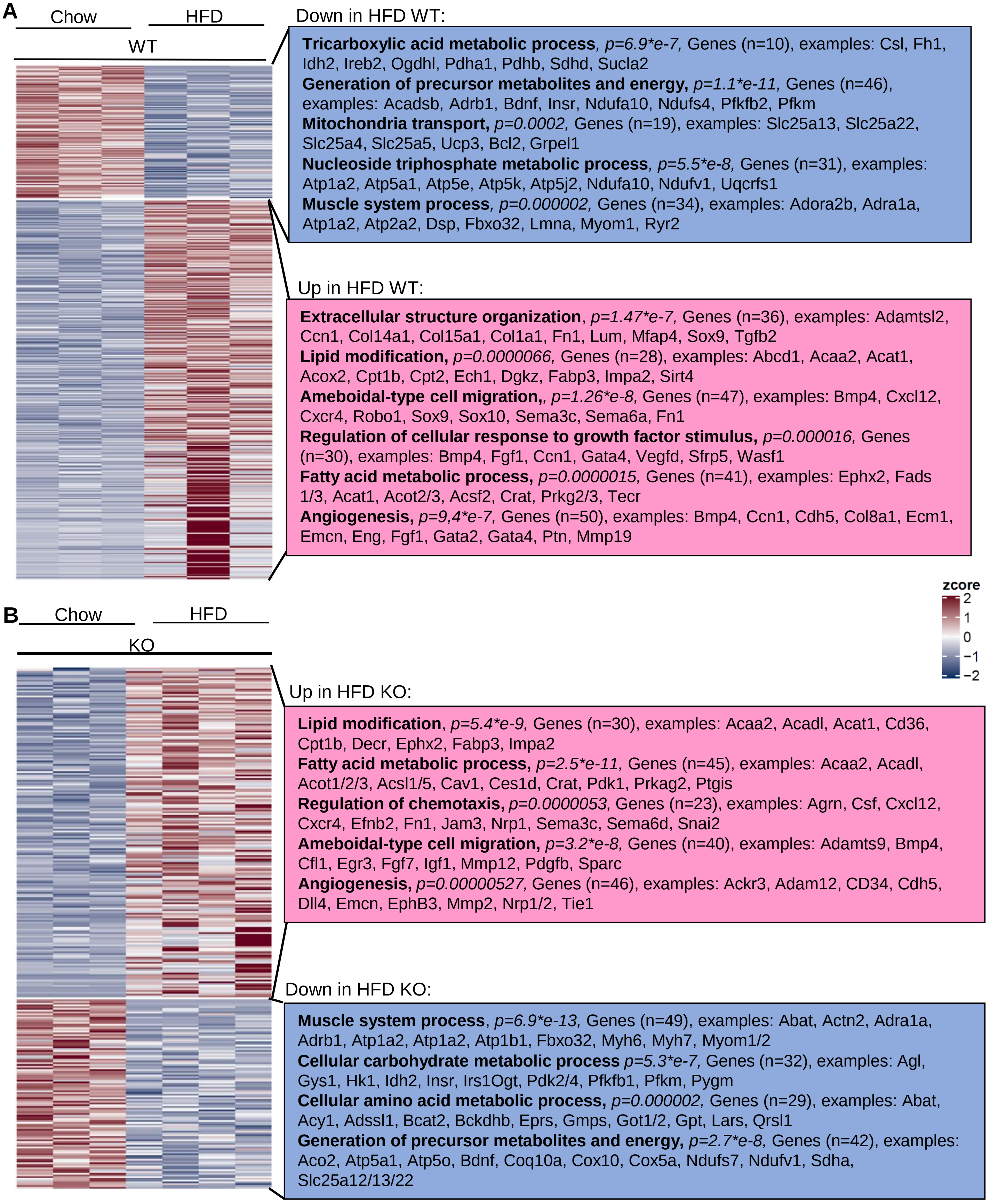

Supplemental Figure S5. Different gene-expression patterns in response to HFD (high fat diet) inCTRP9 knock-out (KO) and wild-type (WT) mice. (A) Transcriptomic profiling in WT mice exposed to 12 weeks of chow or HFD. A heat map of differentially expressed genes is shown. Gene-ontology classes of differentially downregulated and upregulated genes and example genes are shown on

the right. (B) Transcriptomic profiling in KO mice exposed to 12 weeks of chow or HFD. A heat map of differentially expressed genes is shown. Gene-ontology classes of differentially downregulated and up-regulated genes and example genes are shown on the right.
